# Prognostic Biomarkers for Papillary Thyroid Cancer: Reducing Overtreatment, Improving Clinical Efficiency, and Enhancing Patient Experience

**DOI:** 10.1177/15330338251361633

**Published:** 2025-07-31

**Authors:** Oliver F Bathe, Cynthia Stretch

**Affiliations:** 1 Arnie Charbonneau Cancer Research Institute, Cumming School of Medicine, 2129 University of Calgary, Calgary, AB, Canada; 2 Departments of Surgery and Oncology, 2129 University of Calgary, Calgary, AB, Canada; 3 Qualisure Diagnostics Inc., Calgary, AB, Canada

**Keywords:** thyroid cancer, biomarker, prognosis, genome, transcriptome

## Abstract

Papillary thyroid cancer (PTC), the most prevalent form of thyroid malignancy, is generally indolent but poses a recurrence risk of 10%-15%, leading to a clinical paradox: the need to mitigate recurrence while avoiding overtreatment. Current prognostic frameworks, reliant on anatomical and histopathological factors, often result in inefficient treatment pathways, unnecessary surgical interventions, and increased patient burden. The advent of molecular diagnostics presents a paradigm shift in risk stratification. Implementing preoperative molecular tests could transform PTC management by enabling tailored therapeutic strategies, reducing the need for completion thyroidectomies, optimizing the selection of patients for active surveillance, and refining the use of adjuvant therapies such as radioactive iodine. While genomic alterations such as *BRAF* and *TERT* mutations have been explored as prognostic markers, their predictive utility remains limited. In contrast, transcriptomic profiling has emerged as a powerful tool for identifying aggressive PTC subtypes with greater precision. Transcriptomic-based prognostic tests, like the novel Thyroid GuidePx^®^ classifier, effectively stratify PTCs into distinct molecular subgroups with differing recurrence risks, surpassing traditional clinicopathological models in predictive accuracy. By shifting toward biologically informed decision-making, we can enhance clinical efficiency, minimize patient morbidity, and improve overall healthcare resource utilization.

Thyroid cancer is the eighth most common malignancy, with papillary thyroid cancer (PTC) accounting for approximately 80% of cases. While PTC is often described as a “good cancer” due to its typically indolent nature, this characterization can be misleading. For patients, any cancer diagnosis carries significant emotional and clinical implications, necessitating thoughtful and individualized treatment decisions. Structural recurrence occurs in 10%-15% of cases, either locally in the neck or at distant sites, increasing treatment complexity, costs, and impacting quality of life. Therefore, for patients with a high recurrence risk, it is critical for physicians to recommend interventions that effectively mitigate this risk. Conversely, unnecessarily aggressive treatments in low-risk patients may lead to avoidable complications, increased costs, and diminished well-being. Striking this balance is particularly important as the incidence of PTC continues to rise, largely driven by the increased detection of small, early-stage tumors.^1^

Given these challenges, accurate prognostication is essential to inform clinical decision-making. However, current approaches remain inadequate, relying on static clinicopathological factors that fail to capture the biological complexity of individual tumors. The aim of this review is to synthesize current evidence on molecular prognostic markers in PTC, including both mutation-based and transcriptomic-based classifiers, to inform preoperative and postoperative clinical decision-making.

## The Current Standard of Care

### Preoperative Decisions

**Figure 1.**
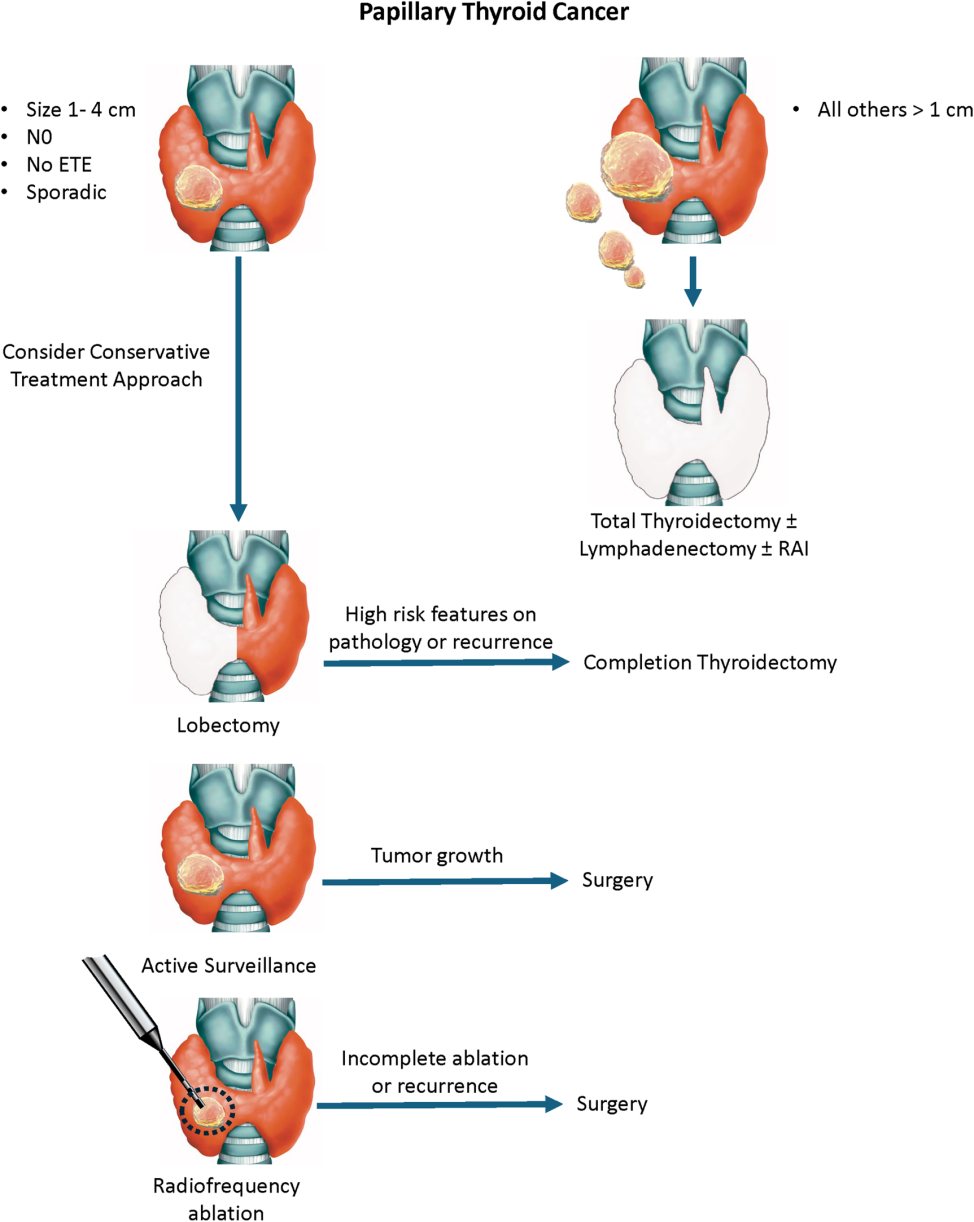
The current treatment pathway for sporadic PTC. Using the current approach, the prognosis is constantly being re-evaluated during treatment, resulting in potential changes in the initial treatment plan. The role of radiofrequency ablation remains undefined.

The current preoperative decision-making framework for papillary thyroid cancer (PTC) relies heavily on anatomical factors, such as tumor size and lymph node involvement (Figure 1). Prognosis is not currently a major consideration when deciding on initial management.

Total thyroidectomy is indicated for bilateral multifocal disease and large tumors, and lymphadenectomy is performed in cases of nodal disease. The role of routine central neck dissection is controversial.^2^ For PTCs measuring 1-4 cm without lymph node involvement or gross extrathyroidal spread, lobectomy (partial thyroidectomy) is an option. The choice between total thyroidectomy and lobectomy is influenced by surgeon preference and patient-specific factors. Some patients may be inclined to pursue a more aggressive approach due to anxiety related to their diagnosis. Older individuals and those with comorbidities may benefit from less extensive interventions.

For small tumors (<1.5-2 cm) confined to the thyroid gland, active surveillance is an option,^3^ particularly for older patients or those with comorbidities. In younger individuals, however, active surveillance requires extended follow-up and may not be suitable for those prone to anxiety regarding their diagnosis. Another emerging approach is radiofrequency ablation (RFA), which has shown promise in the management of benign thyroid nodules and is now being explored for malignant lesions.^4,5^ Complete ablation requires careful patient selection to ensure tumor eradication while minimizing collateral damage. Ongoing clinical trials are evaluating long-term outcomes in selected populations.

Preoperative decisions are not inconsequential. Opting for a more aggressive treatment exposes patients to surgical risks. Total thyroidectomy necessitates lifelong thyroid hormone replacement therapy, which entails periodic dosage adjustments and potential symptoms of hypothyroidism or hyperthyroidism. Additionally, there is a risk of temporary or permanent hypoparathyroidism, as well as injury to one or both recurrent laryngeal nerves. Neck dissections further increase the risk of complications. Conversely, choosing a conservative approach may increase recurrence risk or necessitate additional surgical intervention, as discussed below.

### Postoperative Decisions

Following surgery, the histopathological features of the PTC are examined to refine risk assessment. The American Thyroid Association (ATA) Risk Stratification System categorizes patients based on clinical and pathological features to estimate recurrence risk and guide adjuvant therapy decisions.^6^

In cases where high-risk features are identified after a lobectomy, a completion thyroidectomy may be necessary. However, completion thyroidectomy is technically more challenging and associated with higher rates of complications, including vocal cord paresis (3.3-5.4%), transient hypocalcemia (8.2-9%), and persistent hypocalcemia (0.7-1.5%).^7^ These rates are comparable to those seen with initial total thyroidectomy.^8^

For patients who undergo total or completion thyroidectomy, the decision to administer adjuvant radioactive iodine (RAI) is also based on recurrence risk. The 2015 ATA guidelines recommend RAI for high-risk PTCs, while suggesting a case-by-case approach for intermediate-risk cases.^6^ However, the benefit of RAI in intermediate-risk patients remains debated,^9^ as not all patients with intermediate-risk PTCs benefit.^10^ Furthermore, RAI is inconvenient and has clinical consequences. RAI requires temporary isolation due to radiation exposure. Fertility may be affected, which is particularly pertinent since PTC is often diagnosed at a young age. Pain and swelling from inflammation of the salivary glands, dry mouth, changes in taste, and malaise are not uncommon. Secondary malignancies that emerge years after treatment are also a risk.^11^

## Limitations and Inefficiencies of Current Prognostic Approaches in Thyroid Cancer Treatment

The current approach of using the ATA risk stratification system to estimate recurrence risk has important limitations. Although validated in retrospective studies, its predictive accuracy is suboptimal, explaining only 19%-34% of variance in recurrence outcomes.^12,13^ The system relies on subjective pathological assessments, and the effect size of various factors is not well defined. More critically, prognostication occurs **only after surgery**, meaning high-impact treatment decisions are made without definitive risk assessment.

Several histological features that inform risk classification are inherently subjective and prone to inter-observer variability.^14-16^ Vascular invasion, for example, is not consistently reported among pathologists,^17^ and its clinical significance remains debated. Some studies associate vascular invasion with worse disease-free survival,^18-20^ while others do not find it to be an independent risk factor for recurrence.^21,22^ It is possible that this inconsistency may be attributable to intratumoral heterogeneity, where one tumor section may exhibit vascular invasion while another does not. This highlights the challenges of relying on pathology alone for prognostication.

Aggressive histological variants, such as the tall cell, columnar cell, and hobnail variants, are associated with worse outcomes. The tall cell variant, found in 6%-13% of cases,^23,24^ is the most studied. Historically, there has been debate over the proportion of tall cells required to classify a tumor as a tall cell variant. The current WHO definition specifies a threshold of 30%, yet few studies have systematically examined how different proportions influence recurrence risk.^25,26^ Other high-risk histological subtypes are rare,^24,27^ making their independent prognostic value difficult to establish due to small sample sizes.

The intermediate-risk category within the ATA system is especially troublesome due to its heterogeneity.^28^ Some patients classified as intermediate-risk experience excellent long-term outcomes, while others exhibit recurrence rates more in line with high-risk groups. To address this, the latest ATA guidelines propose subclassifying the intermediate category into low-intermediate and high-intermediate risk. The clinical impact of this change remains to be seen. Moreover, the presence of vascular invasion or an aggressive histologic variant is among the criteria that distinguish intermediate-high and intermediate-low PTCs in the new ATA risk classification system.

It has been suggested that the limited accuracy of the ATA clinical risk stratification system is also a function of its static nature. Tuttle et al reported that recurrence risk is dynamic, modified by treatment response.^12^ This was especially apparent in ATA intermediate-risk patients, where the recurrence rate was as low as 2% in individuals with an excellent response.

What is particularly problematic is that the information required for accurate prognostication using clinicopathologic factors is not available until **after** surgery. This is especially relevant for patients undergoing lobectomy. Multiple series have reported that high-risk features are identified during surgery or postoperatively (in the final pathology) in 40%-60% of patients initially deemed low-risk based on preoperative clinical criteria.^29-34^ Completion thyroidectomies are therefore indicated in a significant proportion of patients who had a lobectomy. This two-step approach nearly doubles treatment costs, prolongs the care timeline, and increases surgical risk. Related to this, patients informed of a potential need for a second surgery often opt for an initial total thyroidectomy to avoid uncertainty, even if their cancer is ultimately low-risk.

Similarly, patients selected for active surveillance or radiofrequency ablation (RFA) are typically chosen based on tumor size alone, without a full understanding of their tumor's biological behavior. Because pathological features and molecular characteristics remain unknown at the time of decision-making, accurately predicting long-term outcomes for these patients remains challenging.

Overall, the current reliance on clinicopathological factors leads to overtreatment. More patients undergo total thyroidectomy than necessary, completion thyroidectomy rates remain unacceptably high, and radioactive iodine (RAI) may be used too frequently. This approach not only results in excessive costs but also negatively impacts patient quality of life due to unnecessary interventions.

A more effective strategy would incorporate molecular profiling **preoperatively** to guide the entire treatment pathway from the outset. Tumor biology is the primary determinant of recurrence risk, yet the current framework relies on pathology-based risk assessment, which only correlates indirectly with biological behavior. Molecular testing is key to addressing this gap, enabling more precise risk stratification, and ensuring that treatment intensity is appropriately matched to individual patient risk.

## Mutations and Gene Fusions as Prognostic Biomarkers

Molecular testing for thyroid nodules initially focused on distinguishing benign from malignant nodules, particularly in cases with indeterminate cytology (Bethesda III and IV). This approach aimed to reduce unnecessary surgeries by identifying patients unlikely to have cancer. Several diagnostic tests, including ThyroSeq^®^, Afirma^®^, ThyraMIR^®^/ThyGeNEXT^®^, ThyroidPrint^®^, and ThyroSPEC™, have proven effective in this setting.^35-39^ However, their role in patients already diagnosed with papillary thyroid cancer (PTC) (Bethesda V/VI) remains undefined.

Historically, efforts to predict PTC prognosis have centered on identifying specific mutations and gene fusions, among which the *BRAF^V600E^* mutation is the most prevalent. The presence of the *BRAF^V600E^* mutation was once considered prognostic,^40,41^ and in animal models, the *BRAF^V600E^* mutation can incite dedifferentiation and resistance to radioactive iodine.^42^ However, subsequent studies have shown conflicting results regarding its impact on recurrence risk, with some reports questioning its utility as an independent prognostic marker.^43-47^ Additionally, the high prevalence of *BRAF^V600E^* in PTC—approximately 50%—limits its discriminative power in stratifying risk.

*TERT* promoter mutations, found in 7%-10% of PTCs,^48-50^ have been more consistently associated with aggressive disease features and increased recurrence rates.^41,45,48,51,52^ Retrospective studies have demonstrated that *TERT* mutations correlate with poor clinical outcomes, particularly in the presence of co-occurring *BRAF^V600E^* or *RAS* mutations. However, their prognostic impact appears diminished when found in isolation, highlighting the complexity of molecular interactions in thyroid cancer.^53^

Chromosomal rearrangements in the *RET* proto-oncogene occur in approximately 4%-7% of adult PTC cases.^49,54^ While tumors harboring *RET* fusions often exhibit aggressive features such as extrathyroidal extension,^54^ their direct influence on structural recurrence remains inconclusive. This underscores the need for additional research to clarify the clinical significance of fusion-driven oncogenesis in PTC.

A recent study explored the potential of using ThyroSeq^®^ to assess PTC prognosis. In that retrospective study of 105 patients, PTCs harboring both *BRAF^V600E^* and *TERT* promoter mutations had a markedly increased recurrence risk.^55^ However, the study's clinical applicability is limited by the fact that the majority of patients—84%—were classified as intermediate-risk, complicating treatment decisions.

Beyond *BRAF^V600E^* and *TERT* promoter mutations and *RET* fusions, PTC harbors a range of other genetic alterations, including *TP53* mutations, *EIF1AX*, *PAX8-PPARG* fusions, and other chromosomal rearrangements.^49^ However, their clinical utility in assessing prognosis is limited and variable. This is particularly due to the low frequency of these alterations in PTC.^49^

**Figure 2.**
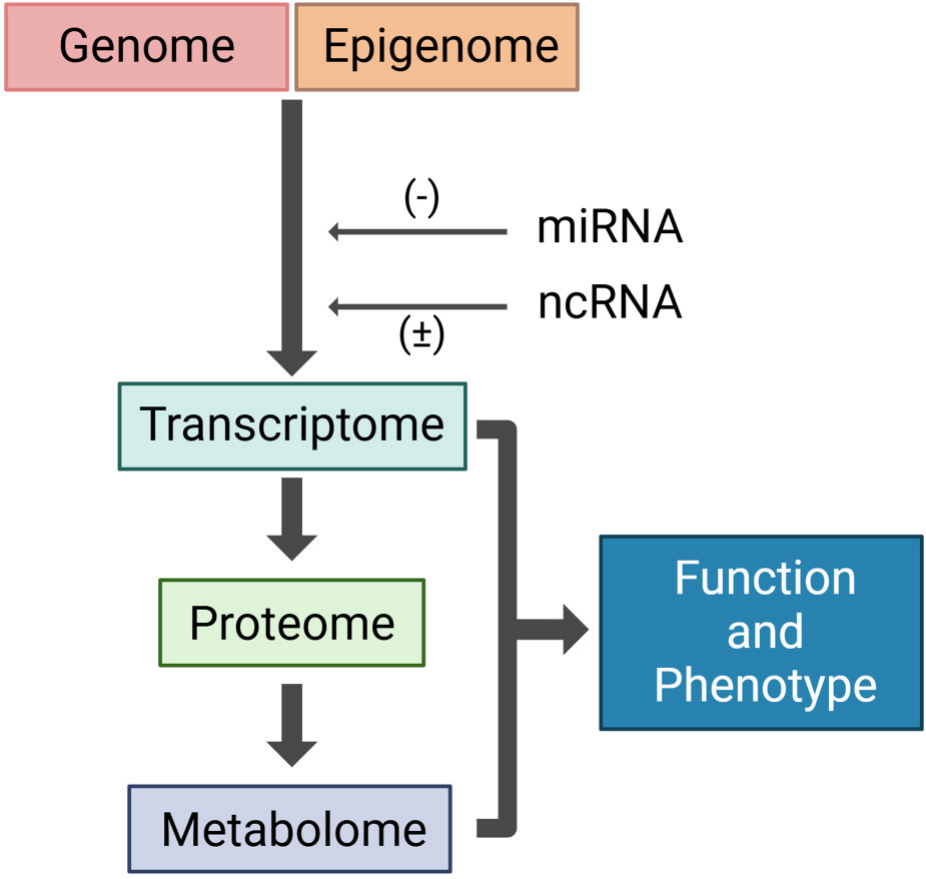
Biological pathway leading to phenotype and function. The genome is hard coded, consisting of gene variants, fusions, copy number variants and single nucleotide variants. Environmental factors affecting the epigenome modify genomic signaling, affecting gene expression. Further modifications of gene expression (mRNA) are influenced by non-coding RNAs (ncRNA) and microRNAs (miRNAs). Gene expression directly affects protein translation and alterations in the metabolome. The transcriptome, proteome and the metabolome are the closest molecular representations of cell phenotype. Created with BioRender.

While genomic technologies such as next-generation sequencing (NGS) have enhanced our ability to detect somatic mutations with high sensitivity, reliance on DNA-based alterations alone presents challenges. Mutations represent the fundamental genetic code (Table 1), but multiple layers of regulation—such as DNA methylation, non-coding RNAs, microRNAs, and alternative splicing—can modulate gene expression and tumor behavior (Figure 2). Furthermore, different mutations within the same gene may exert opposing functional effects, complicating prognostic interpretations. The net functional output of combinations of mutations is also poorly understood.

**Table 1.** Omics technologies used for biomarker discovery: advantages and disadvantages of each type of molecular test.

Omics Technology	Advantages	Disadvantages
Genomics Includes: Mutations Gene fusions Single nucleotide polymorphisms (SNPs) Copy number variations (CNVs)	The genome can be comprehensively evaluated by NGS. NGS is sensitive and specific.	The epigenome and downstream events modify the effects of the genome on function and phenotype. Therefore, the effects of mutations, fusions, SNPs, and CNVs are not entirely predictable. The genome does not change with environmental conditions.
Epigenomics Includes: DNA methylation Histone modifications Chromatin accessibility	The epigenome can be comprehensively evaluated by ChIP-Seq, bisulfite sequencing, ATAC-Seq, and Hi-C.	Comprehensive evaluation requires multiple technology platforms. Downstream events modify the effects on function and phenotype.
Transcriptomics Includes: Gene expression levels (mRNA), including splice variants Gene fusions miRNA and non-coding RNA	The transcriptome can be comprehensively evaluated by NGS. NGS is quantitative and accurate. Gene expression is dynamic. It is a culmination of genomic and epigenomic features, effects of miRNA and non-coding RNAs, and environmental factors. Patterns of gene expression have a direct relationship to function and phenotype. The functional effects of patterns of gene expression are well described.	Sufficient high-quality RNA in a sample is essential. RNA is susceptible to degradation.
Proteomics Includes: Protein identification and quantification Post-translational modifications Protein interactions	Proteomic analysis is quantitative. Proteomic features are a very close reflection of function and phenotype.	The proteome cannot be comprehensively evaluated. There are multiple translational modifications with unknown functions.
Metabolomics Includes: Metabolite abundance	Metabolomic features are a very close reflection of function and phenotype. The metabolome is dynamic and highly responsive to environmental conditions.	The metabolome cannot be comprehensively evaluated. Metabolite biomarkers are very sensitive to sample conditions. The functional effects of specific patterns of metabolites are not well understood.

Abbreviation: NGS, next-generation sequencing.

A key limitation of mutation-based prognostication is the lack of information on the prevalence of these genetic alterations within a tumor. Thyroid cancers, like most malignancies, exhibit significant intratumoral heterogeneity, meaning that not all tumor cells carry the same mutations. Variant allele frequency (VAF), which quantifies the proportion of tumor cells harboring a specific mutation, has been proposed as a measure of prognostic significance. However, VAF remains susceptible to technical biases,^56^ and its clinical utility in PTC remains uncertain.

Given these limitations, there is a pressing need to move beyond DNA-based markers and incorporate broader molecular approaches that capture **functional tumor biology**. Transcriptomic analysis, which examines gene expression profiles, offers a more comprehensive view of tumor behavior and has demonstrated superior prognostic accuracy in PTC. By integrating transcriptomic signatures with clinical factors, we can refine risk stratification and improve personalized treatment strategies for thyroid cancer patients.

## Prognostication Based on the Transcriptome

Next-generation sequencing (NGS) techniques such as RNA sequencing (RNA-Seq) allow for comprehensive transcriptome profiling, capturing gene expression patterns and identifying gene fusions. Unlike DNA-based mutation detection, transcriptomic analysis provides a functional readout of tumor biology, revealing upregulated and downregulated pathways that cannot be inferred solely from mutational status. This integrative approach accounts for multiple upstream influences, including copy number variations, epigenomic modifications, microRNA activity, and noncoding RNA interactions, offering a more nuanced understanding of tumor behavior than static genomic alterations alone.

**Figure 3.**
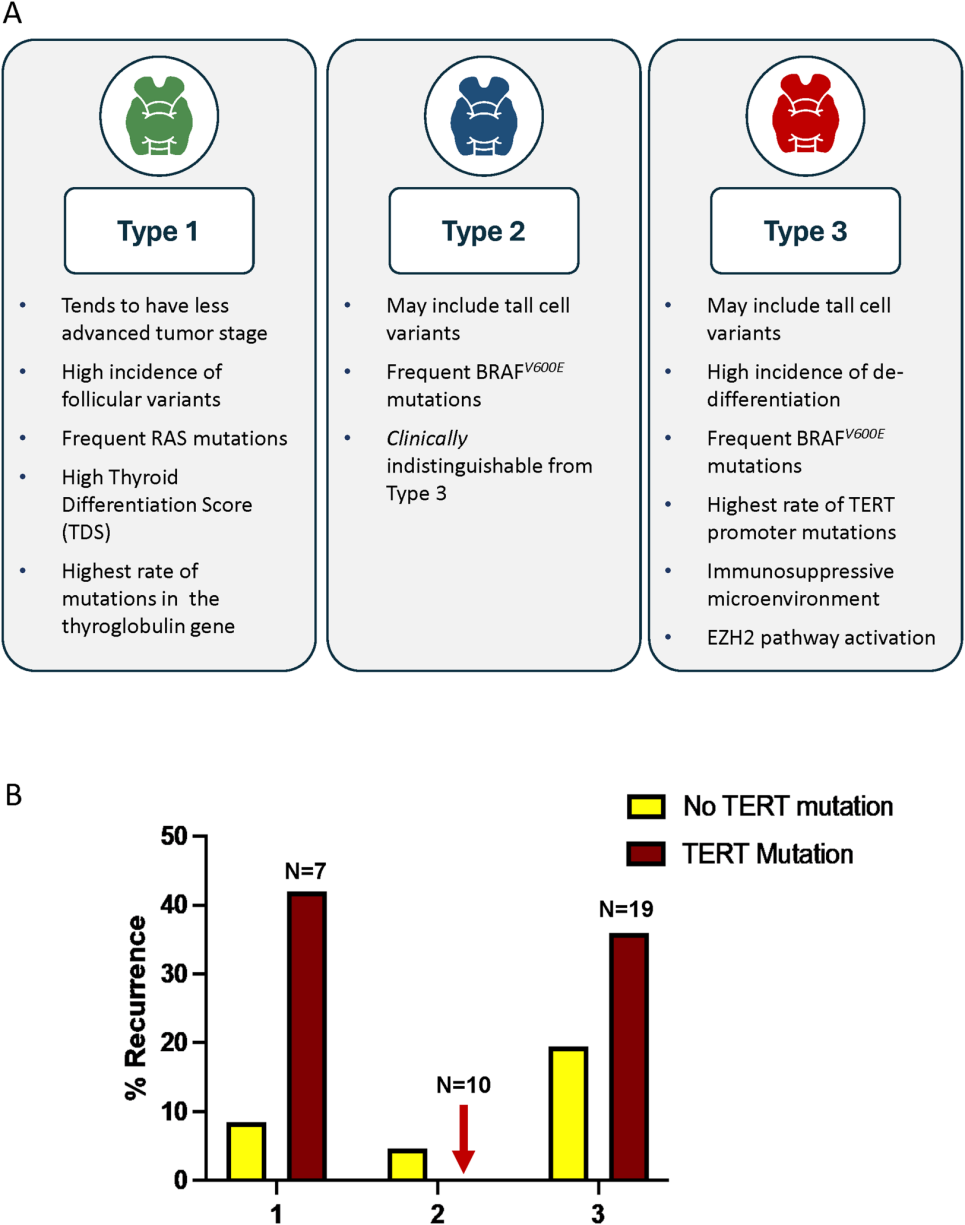
Molecular subtypes identified by Thyroid GuidePx^®^. A. Clinicopathological features of the three molecular subtypes. B. Recurrence rate at 5 years for PTCs with and without *TERT* promoter mutations based on molecular subtype. The data are derived from 502 patients annotated by The Cancer Genome Atlas.^43,49^

To date, the only published biomarker utilizing the transcriptome designed for the purpose of PTC prognostication is Thyroid GuidePx^®^.^43^ This biomarker classifies PTCs into three distinct molecular subtypes with unique clinical and pathological characteristics (Figure 3A). **Type 1 PTCs** were well-differentiated, exhibited low lymph node metastasis rates, and had a minimal risk of recurrence. The majority of follicular variant PTCs were classified as Type 1, with no cases of tall cell variant PTC in this group. Tumors harboring *NRAS* and *HRAS* mutations were predominantly Type 1. **Type 3 PTCs**, in contrast, demonstrated a dedifferentiated phenotype, frequent extrathyroidal extension, and high rates of lymph node metastases and recurrence. They were comprised mostly of classical variants, but 10.8% were tall cell variants. About half had *BRAF^V600E^* mutations, and 13.9% had *TERT* mutations. Type 3 PTCs had the highest structural recurrence rate. **Type 2 PTCs** exhibited clinical and pathological features similar to Type 3 but had significantly lower recurrence rates.

The prognostic molecular subgroups were identified in 335 patients annotated by TCGA. The transcripts influencing structural recurrence were identified by a machine learning algorithm that ranked genes by their prognostic effects, then an unsupervised analysis clustered individuals based on gene expression patterns. The prognostic value of Thyroid GuidePx^®^ was validated across three independent cohorts from the United States (*N* *=* 167), Canada (*N* = 136), and South Korea (*N* = 124).^43^ Consistently, Type 3 PTCs exhibited a high recurrence risk, even among early-stage cases (≤4 cm with no detectable lymph node involvement), with a recurrence rate of approximately 20%. Thyroid GuidePx^®^ demonstrated superior prognostic accuracy, outperforming the ATA system in predicting recurrence, with molecular risk classification proving more predictive than any single clinical or pathological feature aside from distant metastases. Further transcriptomic analyses of available RNA-Seq data revealed biological distinctions between subtypes. Most strikingly, Type 3 PTCs had enrichment of the EZH2-HOTAIR pathway, which is known to induce de-differentiation and can influence tumor immunity. Correspondingly, Type 3 PTCs had an immunosuppressed microenvironment.

Since its initial validation, a recent study evaluated the performance of the Thyroid GuidePx^®^ model in tall cell variant PTCs (*N* = 44), confirming its ability to differentiate high-risk from low-risk cases.^57^ Notably, none of the early-stage Type 2 tall cell variant PTCs experienced recurrence. Likewise, Type 2 PTCs with *TERT* promoter mutations in the TCGA cohort had no recurrences, even if there was a coincidental *BRAF^V600E^* mutation (Figure 3B).

## Prognostic Tests to Enhance Clinical Care

**Figure 4.**
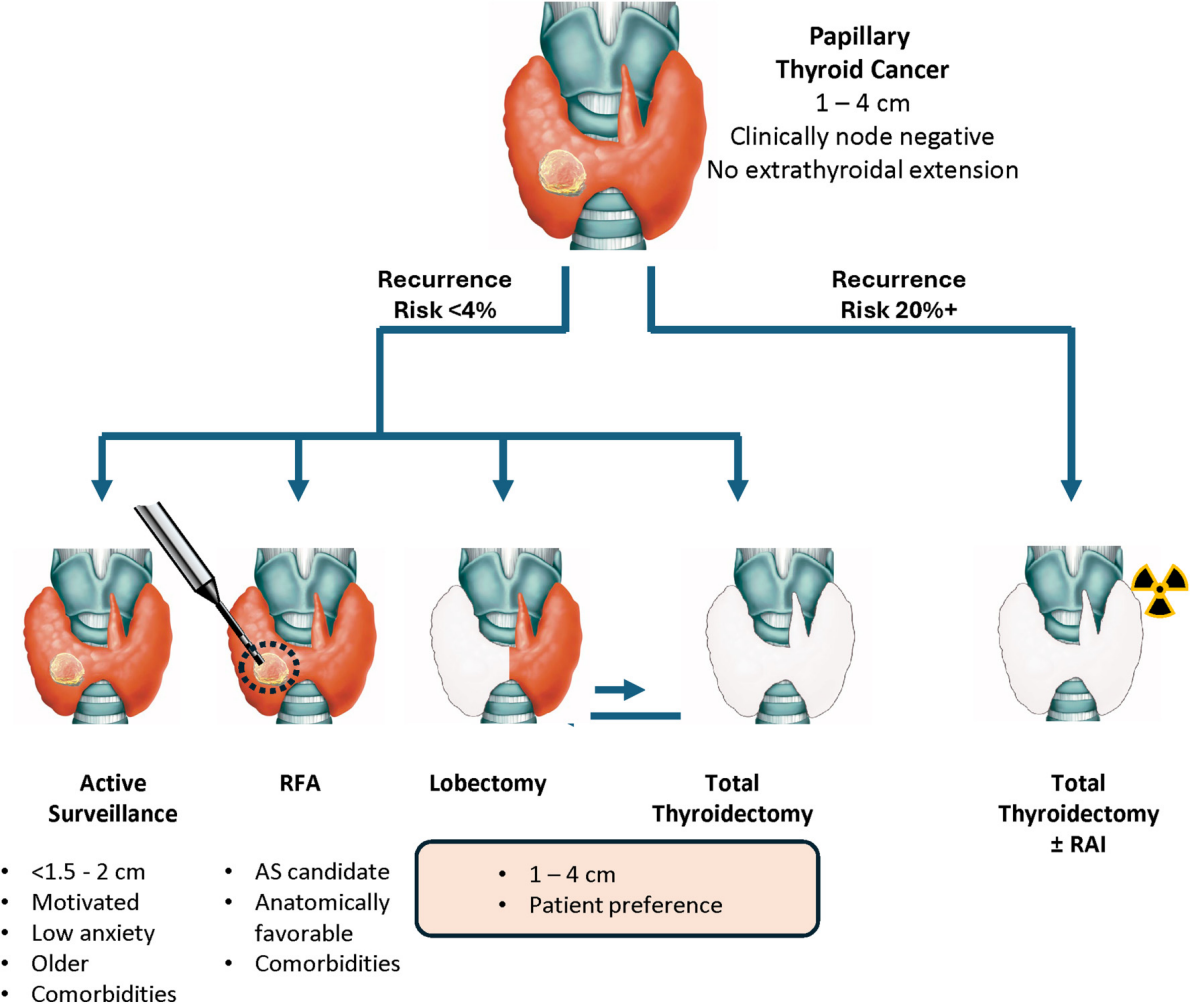
Making decisions with the prognostic information derived from Thyroid GuidePx^®^. Recognizing high risk cancers preoperatively reduces the need for completion thyroidectomies. Potentially, patients informed of a very low risk of recurrence will tend toward more conservative procedures because they increased confidence in their prognosis and because they are less likely to require a completion thyroidectomy.

Incorporating molecular testing into the preoperative setting has the potential to transform the entire treatment pathway for PTC. This is particularly impactful when evaluating candidates for conservative management strategies such as lobectomy, RFA, or active surveillance (Figure 4). Patients with solitary, intrathyroidal PTCs up to 4 cm and no lymph node involvement would significantly benefit from preoperative molecular stratification. By identifying low-risk tumors, prognostic tests could facilitate more confident decision-making regarding conservative treatment approaches, reducing unnecessary total thyroidectomies. Conversely, early identification of high-risk PTCs could appropriately direct these patients toward total thyroidectomy and more intensive follow-up. For the physician who must present nuanced treatment options to the patient, a molecular test that conveys a very low risk or a high risk of recurrence would simplify the discussion.

One potential impact of prognostic tests is their influence on lobectomy and completion thyroidectomy rates. A significant proportion of patients who undergo lobectomy ultimately require a second surgery due to the identification of high-risk features in postoperative pathology. Preoperative molecular classification would allow for better-informed surgical planning, reducing the need for completion thyroidectomy and minimizing patient anxiety over treatment escalation. Additionally, many patients opt for total thyroidectomy upfront due to the uncertainty of postoperative findings. A reliable molecular risk assessment tool could provide greater confidence in selecting a lobectomy when appropriate, thereby improving patient experience and reducing unnecessary interventions.

Active surveillance for PTC is gaining acceptance as long-term studies confirm its safety. However, its cost-effectiveness remains unclear,^58,59^ and psychological distress associated with prolonged monitoring may impact quality of life.^60-62^ Molecular recurrence risk assessment could refine patient selection, ensuring that only those with the lowest recurrence risk are enrolled in surveillance programs. This could reduce the need for intensive follow-up imaging and biopsies, thereby enhancing both cost-effectiveness and patient reassurance.

The role of RFA in PTC treatment continues to evolve, with promising early results. Successful outcomes depend heavily on proper patient selection, making molecular recurrence risk an important consideration. Further studies are needed to establish how transcriptomic classification can optimize patient selection for RFA and other emerging treatment modalities.

Molecular testing also has implications for patients requiring total thyroidectomy. Following surgery, recurrence risk assessment can guide decisions regarding adjuvant radioactive iodine therapy. This is particularly valuable in intermediate-risk PTC, where the benefit of RAI remains uncertain. A robust molecular classifier such as Thyroid GuidePx^®^ could refine RAI selection, ensuring its use is reserved for those most likely to benefit, thereby reducing unnecessary radiation exposure and associated long-term side effects.

Beyond guiding initial treatment decisions, prognostic tests may also influence post-treatment surveillance strategies. High-risk patients could be prioritized for more intensive follow-up protocols, while those classified as low-risk may require less frequent monitoring. This personalized approach to post-treatment care would optimize resource utilization while reducing patient burden.

Finally, the economic implications of molecular prognostication should not be overlooked. Thyroid cancer has been associated with significant financial toxicity.^63,64^ Of all cancers, thyroid cancer is responsible for the highest rate of bankruptcy.^65^ This is especially pertinent since thyroid cancer frequently inflicts persons of working age, especially women. Prolonged treatment timelines, unnecessary surgeries, and excessive surveillance contribute to these economic burdens. By improving risk stratification and streamlining the treatment pathway, prognostic tests like Thyroid GuidePx^®^ could reduce overtreatment, minimize unnecessary interventions, and enhance overall healthcare efficiency. Cost savings would be realized through fewer total thyroidectomies, fewer completion thyroidectomies, reduced reliance on RAI, and improved patient selection for non-surgical options such as RFA and active surveillance**.**

As precision medicine continues to evolve, molecular prognostication represents the next frontier in thyroid cancer management**.** While future prospective studies and rigorous retrospective analyses are needed to address questions of accessibility, cost-effectiveness, and standardization, prognostic tests have the potential to redefine risk assessment, enabling truly personalized treatment strategies that optimize patient outcomes while reducing unnecessary medical interventions.

## Conclusion

Current PTC management is constrained by imprecise risk stratification models that rely on static clinicopathological variables. The advent of transcriptomic-based prognostication, exemplified by Thyroid GuidePx^®^**,** represents a major advancement in personalized thyroid cancer care. By incorporating molecular risk assessment into preoperative decision-making, clinicians can optimize treatment pathways, minimize overtreatment, and improve long-term patient outcomes. As molecular diagnostics continue to evolve, their integration into clinical practice will be essential in refining the standard of care for PTC patients and advancing the principles of precision oncology.
